# Association between the stress–hyperglycemia ratio and all‐cause mortality in community‐dwelling populations: An analysis of the National Health and Nutrition Examination Survey (NHANES) 1999–2014

**DOI:** 10.1111/1753-0407.13567

**Published:** 2024-05-20

**Authors:** Shifeng Qiu, Xiaocong Liu, Li Lei, Hongbin Liang, Xue Li, Yutian Wang, Chen Yu, Xiaobo Li, Yongzhen Tang, Juefei Wu, Yuegang Wang, Daogang Zha, Xuewei Liu, Min Xiao, Jiancheng Xiu

**Affiliations:** ^1^ Department of Cardiology Nanfang Hospital, Southern Medical University Guangzhou China; ^2^ Guangdong Provincial Key Laboratory of Shock and Microcirculation Nanfang Hospital, Southern Medical University Guangzhou China; ^3^ State Key Laboratory of Organ Failure Research Nanfang Hospital, Southern Medical University Guangzhou China; ^4^ Department of Cardiology Shenzhen People's Hospital (The Second Clinical Medical College, Jinan University, The First Affiliated Hospital, Southern University of Science and Technology) Shenzhen China; ^5^ Department of Gastroenterology Nanfang Hospital, Southern Medical University Guangzhou China; ^6^ Department of Cardiology Xiangdong Hospital Affiliated to Hunan Normal University Zhuzhou China; ^7^ Department of General Practice Nanfang Hospital, Southern Medical University Guangzhou China; ^8^ The First School of Clinical Medicine Southern Medical University Dongguan China

**Keywords:** all‐cause mortality, community‐dwelling, NHANES, stress–hyperglycemia ratio, U‐shaped

## Abstract

**Background:**

Reportedly, the stress–hyperglycemia ratio (SHR) is closely associated with poor prognosis in patients with severe acute disease. However, the community‐dwelling may also be in a state of stress due to environmental exposure. Our study aimed to explore the association between SHR and all‐cause mortality in the community‐dwelling population.

**Methods:**

A total of 18 480 participants were included out of 82 091 from the NHANES 1999–2014 survey. The Kaplan–Meier survival analyses were used to assess the disparities in survival rates based on SHR, and the log‐rank test was employed to investigate the distinctions between groups. The multivariate Cox regression analysis and restricted cubic spline (RCS) analysis were performed to assess the association of SHR with all‐cause mortality. A subgroup analysis was also conducted.

**Results:**

A total of 3188 deaths occurred during a median follow‐up period of 11.0 (7.7; 15.4) years. The highest risk for all‐cause mortality was observed when SHR≤ 0.843 or SHR ≥0.986 (log‐rank *p* < .001). After adjusting for the confounding factors, compared with subjects in the second SHR quartile (Q2), participants in the highest (Q4, adjusted hazard ratio [HR] 1.49, 95% confidence interval [CI] 1.28–1.73) and lowest quartiles (Q1, adjusted HR 1.37, 95% CI 1.16–1.60) have a higher probability of all‐cause death. The RCS observed a dose‐response U‐shaped association between SHR and all‐cause mortality. The U‐shaped association between SHR and all‐cause mortality was similar across subgroup analysis.

**Conclusions:**

The SHR was significantly associated with all‐cause mortality in the community‐dwelling population, and the relationship was U‐shaped.

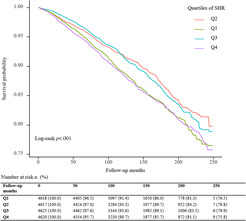

## Graphical abstract

Highlights

- *Based on 18 480 residents, our study found for the first time that the stress–hyperglycemia ratio (SHR) is significantly associated with all‐cause mortality in community dwellers.*
- *SHR and all‐cause mortality had a dose–response relationship, and the relationship was U shaped.*
- *After adjusting for the confounding factors, compared with subjects in the second SHR quartile (Q2), participants in the highest (Q4, adjusted hazard ratio [HR] 1.49, 95% confidence interval [CI] 1.28–1.73) and lowest quartiles (Q1, adjusted HR 1.37, 95% CI 1.16–1.60) have a higher probability of all‐cause death.*

## 1 INTRODUCTION

The stress reaction is the physiological response and psychological response produced by the body to stressors, which is manifested by the rise of blood glucose and blood pressure (BP), emotional reaction, and so on.^1^ In some situations, stress can be reflected in the stress–hyperglycemia ratio (SHR).^2^, ^3^, ^4^ Series studies have confirmed that the SHR is closely associated with poor prognosis in patients with severe acute diseases,^2^ such as myocardial infarction (MI),^5^, ^6^, ^7^, ^8^ stroke,^9^, ^10^ sepsis,^11^ and trauma.^12^ A large cohort study in Asia finds there were U‐shaped associations of SHR with major adverse cardiovascular and cerebrovascular events rate and major adverse cardiovascular events rate at 2‐year follow‐ups and J‐shaped associations of SHR with in‐hospital cardiac death and MI.^13^

However, the rise of blood glucose under stress may not only be a superficial phenomenon of the stress response but also include (a) insulin resistance,^14^, ^15^, ^16^ (b) immune inflammation and endothelial injury,^17^ and (c) psychological factors.^18^ Previous studies have shown that not only the psychological factor is one of the important factors affecting glucose metabolism^19^ but also depression, general emotional stress, anxiety, sleep problems, anger, and hostility are associated with an increased risk of developing type 2 diabetes.^18^

In the community‐dwelling population, the SHR may indicate that the individual is in a state of stress (such as work stress,^20^, ^21^ life stress,^22^ emotional stress,^18^ psychological stress^23^) due to environmental exposure, which could significantly affect the subject's prognosis.^24^, ^25^ However, no studies have investigated the association between SHR and all‐cause mortality in the community‐dwelling population.

Therefore, our study aimed to explore the association between SHR and all‐cause mortality in the community‐dwelling population.

## 2 METHODS

### 2.1 Study population

The National Health and Nutrition Examination Survey (NHANES) is an ongoing cross‐sectional survey to evaluate the health and nutritional status of the US civilian noninstitutionalized population. The NHANES interview includes demographic, socioeconomic, dietary, and health‐related questions, which can be used to determine the prevalence of major diseases and risk factors for diseases. NHANES was approved by the National Center for Health Statistics and all participants provided informed consent.^26^, ^27^

**FIGURE 1:**
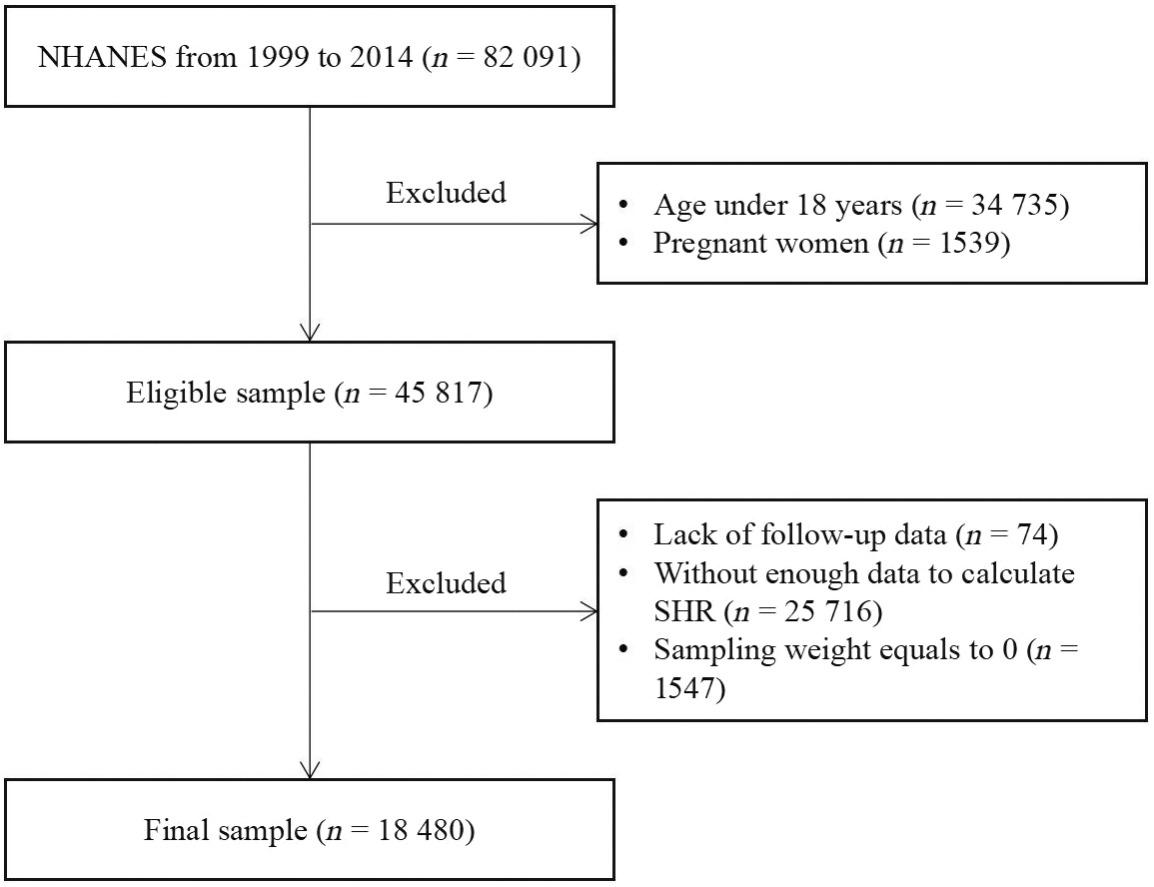
Flow chart of the case selection progress. NHANES, National Health and Nutrition Examination Survey; SHR, stress–hyperglycemia ratio.

The total number of participants from the NHANES 1999–2014 survey is 82 091. We first excluded participants who were < 18 years old or were pregnant. We then further excluded those with no follow‐up data or missing data on components of the SHR, or sampling weight equal to 0. Finally, 18 480 participants were included in our analysis (Figure 1). The NHANES 1999–2014 survey data were obtained from the official website of NHANES. On the website, data are provided every 2 years (a survey cycle). Code books are also provided to help statisticians to understand NHANES files. After downloading the data, we conducted data cleaning and data analysis with R version 4.2.1 software.

### 2.2 Outcomes

The primary outcome of this study was all‐cause mortality. The secondary outcome was cause‐specific death, including death caused by cardiovascular disease (CVD), renal diseases, malignant neoplasms, cerebrovascular diseases, chronic lower respiratory diseases, and Alzheimer's disease. All mortality follow‐up data were derived from the public‐use Linked Mortality Files from the date of survey interview participation through December 31, 2019.^28^ The cause of death was determined using the codes of the *International Classification of Diseases, Tenth Revision*. The codes of CVD mortality were coded I00 to I09, I11, I13, I20 to I51, and I60 to I69. Deaths caused by renal diseases were coded N00‐N07, N17‐N19, and N25‐N27. Malignant neoplasms, cerebrovascular diseases, chronic lower respiratory diseases, and Alzheimer's disease were codes C00‐C97, I60‐I69, J40‐J47, and G30, respectively.

### 2.3 Exposure

The variable being exposed was the SHR. The calculation formula of SHR was^2^, ^4^:

$$\mathrm{SHR}=\left[\text{Acute glucose value}\;\left(\text{mmol}/L\right)\;\right]/\left[1.59\times \mathrm{HbA}1c\;\left(\%\right)-2.59\right]$$

Here use fasting blood glucose (FBG) instead of random blood glucose (RBG). The collection of blood samples and lipid concentration measurements were based on standardized procedures. Glucose value was obtained by the enzyme hexokinase method.^29^ Hemoglobin a1c (HbA1c) was determined by high‐pressure liquid phase ion exchange chromatography.^30^ Laboratory methods are available at http://cdc.gov/nchs/nhanes.

### 2.4 Covariates

The covariates were assessed at baseline by using standardized questionnaires and physical examinations. These covariates included age, sex, body mass index (BMI), waist, systolic blood pressure (SBP), diastolic blood pressure (DBP), ethnicity (Mexican American, Non‐Hispanic Black, Non‐Hispanic White, other Hispanic, other race), education (Less than high school, high school or equivalent, college or above), marital status (married, separated, unmarried), poverty, leisure time physically inactive, never drinking, and tobacco use (never, used to smoke, current smoker). Serum total cholesterol (TC), high‐density lipoprotein cholesterol (HDL‐C), FBG, hemoglobin, HbA1c, alanine transaminase (ALT), and serum creatinine were measured by enzymatic methods, direct immunoassay, kinetic rate method, latex‐enhanced nephelometry, and high‐pressure liquid chromatography, respectively. The estimated glomerular filtration rate (eGFR) was calculated by the Chronic Kidney Disease Epidemiology Collaboration equation.^31^ In addition, poverty was defined as those with household incomes ≤300% of the federal poverty level.^32^ According to the 2008 Physical Activity Guidelines for Americans, leisure time physical activity was divided into inactive and active.^33^ CVD, hypertension, diabetes, and chronic kidney diseases (CKD) were covariates. CVD includes coronary heart disease, angina, heart attack, stroke, and congestive heart failure. Hypertension was defined as blood pressure of more than 140/90 mm Hg or the use of antihypertensive medications. Criteria for the diagnosis of diabetes were: fasting plasma glucose ≥126 mg/dL (7.0 mmol/L); or 2‐h PG ≥200 mg/dL (11.1 mmol/L) during oral glucose tolerance test; or HbA1c ≥6.5% (48 mmol/mol); or in a patient with classic symptoms of hyperglycemia or hyperglycemic crisis, a random PG ≥200 mg/dL (11.1 mmol/L); or use of glucose‐lowering drugs or insulin, or self‐reported diabetes.^34^ CKD was defined as incident eGFR less than 60 mL/min/1.73 m^2^.^35^

### 2.5 Statistical analysis

Values are presented as mean ± SE for continuous variables with normal distribution, medians (interquartile ranges) for nonnormally distributed continuous variables, and number (%) for categorical variables. To detect the differences between groups classified by SHR, linear regression models (continuous variable), and chi‐square tests (categorical variables) were performed. All estimates accounted for sample weights and complex survey designs, and means and percentages were adjusted for survey weights of NHANES.

The Kaplan–Meier survival analyses were used to assess the disparities in survival rates based on SHR, and the log‐rank test was employed to investigate the distinctions between groups. Multivariate Cox regression models were applied to evaluate the association between SHR and all‐cause mortality. Model I was adjusted for age, sex, and ethnicity. Model II was adjusted for age, sex, race/ethnicity, BMI, education levels, marital status, economic status, smoking status, alcohol drinking status, leisure time physically inactive, SBP, DBP, hemoglobin, TC, HDL‐C, uric acid, ALT, eGFR, and self‐reported cardiovascular disease using appropriate sampling weights (fasting subsample weights). Nonlinear relationships were evaluated by multivariable‐adjusted restricted cubic spline (RCS) models with three knots. Additionally, subgroup analysis was conducted based on age (<60 or ≥ 60 years), sex, race/ethnicity (White or non‐White), SBP (<140 or ≥140 mmHg), and FBG (<7.0 or ≥7.0 mmol/L). We applied R version 4.2.1 (R Foundation for Statistical Computing, Vienna, Austria) to perform all the analyses, with *p* < .05 confirmed as statistical significance. The nhanesR package and webpage were used to explore the NHANES database.^36^

## 3 RESULTS

### 3.1 Baseline characteristics

Table 1 presents the characteristics of 18 480 participants in this study. The participants were further divided into four groups and the mean SHR levels were ≤0.843 (Q1: *n* = 4618), 0.844 ~ 0.910 (Q2: *n* = 4617), 0.911 ~ 0.985 (Q3: *n* = 4625), and ≥0.986 (Q4: *n* = 4620) respectively. Compared with residents with lower SHR, residents with higher SHR have a higher proportion of males, married proportion, hemoglobin, ALT level, and uric acid level but a lower proportion of nondrinking (all *p* < .001). Meanwhile, age, race/ethnicity, BMI, education levels, marital status, economic status, smoking status, leisure time physically inactive, and almost all of the laboratory measurements were significantly different among the four groups (all *p* < .001).

**TABLE 1:** Baseline characteristics of the study participants.

n	Overall	Q1 (≤0.843)	Q2 (0.844–0.910)	Q3 (0.911–0.985)	Q4 (≥0.986)	*p* value
	18480	4618	4617	4625	4620	
Age, years	45.7 ± 0.3	47.5 ± 0.4	45.8 ± 0.4	44.3 ± 0.4	45.6 ± 0.3	<.001
Male, *n* (%)	9214 (49.9)	1919 (39.6)	2087 (43.2)	2386 (50.2)	2822 (61.6)	<.001
Pulse, bpm	71.4 ± 0.2	70.9 ± 0.3	71.1 ± 0.2	71.3 ± 0.2	72.2 ± 0.3	<.001
BMI, kg/m^2^	28.4 ± 0.1	28.4 ± 0.1	28.0 ± 0.1	28.3 ± 0.1	29.1 ± 0.1	<.001
Waist, cm	97.3 ± 0.2	96.3 ± 0.3	95.6 ± 0.3	97.0 ± 0.3	100.0 ± 0.4	<.001
SBP, mm Hg	120.7 ± 0.2	121.1 ± 0.4	119.3 ± 0.4	119.8 ± 0.3	122.8 ± 0.4	<.001
DBP, mm Hg	70.2 ± 0.2	69.4 ± 0.3	69.9 ± 0.3	70.1 ± 0.2	71.1 ± 0.2	<.001
Ethnicity, *n* (%)						<.001
Mexican American	3525 (19.1)	709 (6.9)	865 (7.6)	966 (8.7)	985 (8.5)	
Non‐Hispanic Black	3680 (19.9)	1465 (20.4)	918 (11.1)	645 (7.3)	652 (7.3)	
Non‐Hispanic White	8555 (46.3)	1721 (60.1)	2115 (69.3)	2351 (73.3)	2368 (74.1)	
Other Hispanic	1405 (7.6)	360 (5.7)	368 (5.4)	343 (5.2)	334 (4.9)	
Other race	1315 (7.1)	363 (7.0)	351 (6.5)	320 (5.6)	281 (5.2)	
Education, *n* (%)						<.001
Less than high school	5399 (29.3)	1416 (22.0)	1249 (16.9)	1293 (18.6)	1441 (20.1)	
High school or equivalent	4386 (23.8)	1116 (25.0)	1087 (24.2)	1119 (24.4)	1064 (24.0)	
College or above	8668 (47)	2079 (53.0)	2275 (58.9)	2206 (57.0)	2108 (55.9)	
Marital status, *n* (%)						.003
Married	10 442 (58.7)	2553 (62.8)	2585 (63.5)	2655 (64.0)	2649 (64.8)	
Separated	3731 (21)	1055 (20.2)	952 (17.9)	847 (16.2)	877 (16.3)	
Unmarried	3603 (20.3)	859 (17.0)	917 (18.6)	938 (19.8)	889 (18.9)	
Poverty, *n* (%)	10 680 (62.7)	2754 (54.4)	2581 (48.9)	2687 (51.3)	2658 (49.2)	<.001
Leisure time physically inactive, *n* (%)	8643 (46.8)	2292 (44.9)	2120 (40.1)	2038 (39.1)	2193 (41.2)	.001
Never drinking, *n* (%)	3117 (22.5)	884 (22.4)	724 (17.3)	738 (17.1)	771 (16.9)	<.001
Tobacco use, *n* (%)						<.001
Never	9218 (53.6)	2398 (53.7)	2334 (53.4)	2320 (54.1)	2166 (50.8)	
Used to smoke	4379 (25.5)	970 (21.8)	1067 (24.6)	1059 (23.8)	1283 (28.8)	
Current smoker	3594 (20.9)	974 (24.5)	883 (21.9)	892 (22.1)	845 (20.4)	
Laboratory results						
Hemoglobin, g/dl	14.5 (13.5, 15.5)	14.0 (13.1, 15.0)	14.3 (13.4, 15.3)	14.6 (13.7, 15.6)	15.0 (13.9, 15.9)	<.001
TC, mmol/L	5.0 (4.3, 5.7)	5.0 (4.3, 5.8)	5.0 (4.4, 5.7)	4.9 (4.3, 5.6)	4.9 (4.3, 5.6)	<.001
HDL‐C, mmol/L	1.3 (1.1, 1.6)	1.4 (1.1, 1.7)	1.3 (1.1, 1.7)	1.3 (1.1, 1.6)	1.2 (1.0, 1.5)	<.001
Fasting glucose, mmol/L	5.4 (5.1, 5.9)	5.0 (4.7, 5.4)	5.3 (5.0, 5.6)	5.5 (5.2, 5.9)	5.9 (5.5, 6.6)	<.001
HbA1c, %	5.4 (5.1, 5.7)	5.6 (5.4, 5.9)	5.4 (5.2, 5.6)	5.3 (5.1, 5.5)	5.2 (4.9, 5.5)	<.001
ALT, nmol/s/L	21.0 (16.0, 29.0)	20.0 (16.0, 26.0)	20.0 (16.0, 27.0)	21.0 (16.0, 29.0)	23.0 (18.0, 32.0)	<.001
eGFR, mL/min per 1.73 m^2^	97.2 (82.1, 111.2)	95.9 (80.0, 110.5)	96.8 (82.1, 111.4)	98.1 (83.1, 112.1)	97.4 (82.6, 111.0)	.001
Uric acid, μmol/L	321.2 (267.7, 380.7)	309.3 (255.8, 368.8)	315.2 (261.7, 368.8)	321.2 (267.7, 374.7)	339.0 (279.6, 392.6)	<.001
Comorbidities						
CVD	1895 (11.1)	481 (9.2)	452 (8.0)	438 (8.3)	524 (9.5)	.1
Hypertension	7297 (39.5)	1934 (37.4)	1669 (31.6)	1683 (32.9)	2011 (40.0)	<.001
Diabetes	3081 (16.7)	761 (13.6)	462 (7.5)	560 (8.9)	1298 (21.8)	<.001
CKD	1464 (8)	415 (7.4)	328 (5.3)	332 (4.9)	389 (5.9)	<.001

Abbreviations: ALT, alanine transaminase; BMI, body mass index; CKD, chronic kidney diseases; CVD, cardiovascular disease; DBP, diastolic blood pressure; eGFR, estimated glomerular filtration rate; HbA1c, hemoglobin a1c; HDL‐C, high‐density lipoprotein cholesterol; SBP, systolic blood pressure; TC, total cholesterol.

### 3.2 The relationship of SHR with mortality

**FIGURE 2:**
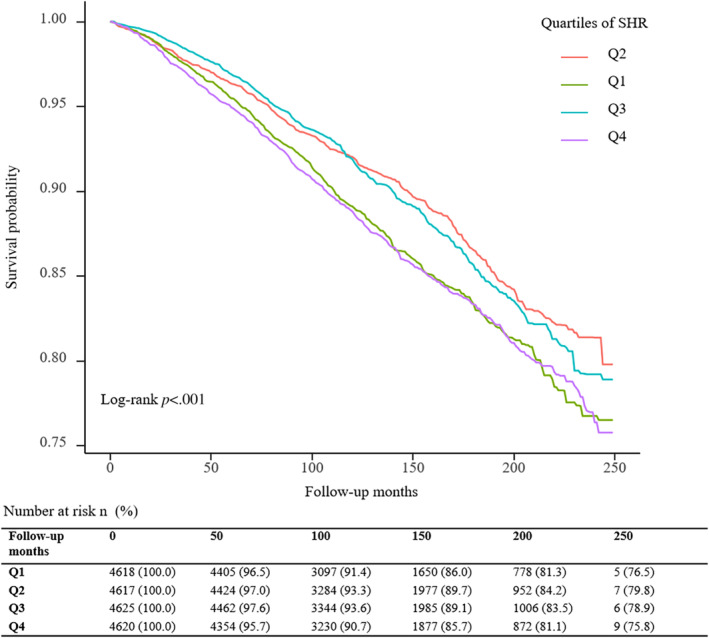
The Kaplan–Meier survival curve and data on survival outcomes for all‐cause mortality by stress–hyperglycemia ratio (SHR).

**FIGURE 3:**
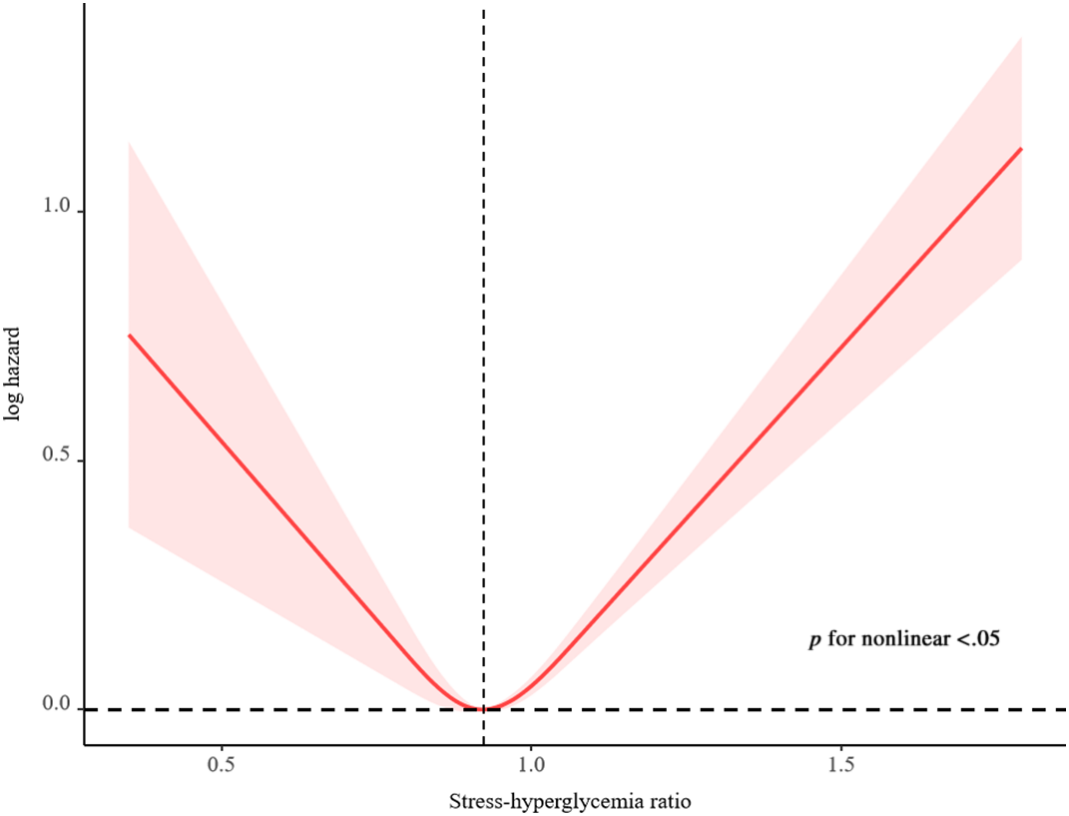
Restricted cubic spline for the association between SHR and all‐cause mortality. Adjusted for age, sex, race or ethnicity, body mass index, education levels, marital status, economic status, smoking status, alcohol drinking status, leisure time physically inactive, systolic blood pressure, diastolic blood pressure, hemoglobin, total cholesterol, high‐density lipoprotein cholesterol, uric acid, alanine transaminase, estimated glomerular filtration rate, and self‐reported cardiovascular disease using appropriate sampling weights.

A total of 3188 deaths (15.0 per 1000 person‐years) occurred during a median follow‐up period of 11.0 (7.7; 15.4) years. The Kaplan–Meier survival curves diverged according to SHR groups (Figure 2). The highest risk for all‐cause mortality and cardiovascular mortality risk was observed when SHR ≤0.843 (Q1) or SHR ≥0.986 (Q4) (log‐rank *p* < .001). The multivariate Cox regression results are demonstrated in Table 2. After adjusting for the confounding factors, compared with subjects in the second SHR quartile (Q2), participants in the highest (Q4, adjusted hazard ratio [HR] 1.49, 95% confidence interval [CI] 1.28–1.73) and lowest quartiles (Q1, adjusted HR 1.37, 95% CI 1.16–1.60) have a higher probability of all‐cause death. Adjusted RCS revealed a U‐shaped association of SHR with all‐cause mortality (Figure 3). The association of SHR with cause‐specific mortality is displayed in Figure **S1** and Table **S1**.

**TABLE 2:** Multivariate Cox regression analysis of SHR with all‐cause mortality.

	Event (%)^a^	Adjusted model 1^b^		Adjusted model 2^c^	
		HR (95% CI)	*p* value	HR (95% CI)	*p* value
All‐cause mortality					
Q1	784 (15.3)	1.18 (1.06–1.32)	.004	1.37 (1.16–1.60)	<.001
Q2	733 (13.6)	Reference		Reference	
Q3	754 (13.8)	1.11 (0.98–1.25)	.097	1.28 (1.09–1.51)	.002
Q4	917 (17.4)	1.26 (1.13–1.40)	<.001	1.49 (1.28–1.73)	<.001

Abbreviations: CI, confidence interval; HR, hazard ratio; SHR, stress–hyperglycemia ratio.

^a^ The event rate was expressed as all‐cause mortality per 1000 person‐years.

^b^ Adjusted for age, sex, race or ethnicity.

^c^ Adjusted for age, sex, race or ethnicity, body mass index, education levels, marital status, economic status, smoking status, alcohol drinking status, leisure time physically inactive, systolic blood pressure, diastolic blood pressure, hemoglobin, total cholesterol, high‐density lipoprotein cholesterol, uric acid, alanine transaminase, estimated glomerular filtration rate, and self‐reported cardiovascular disease using appropriate sampling weights.

### 3.3 Subgroups analysis

**FIGURE 4:**
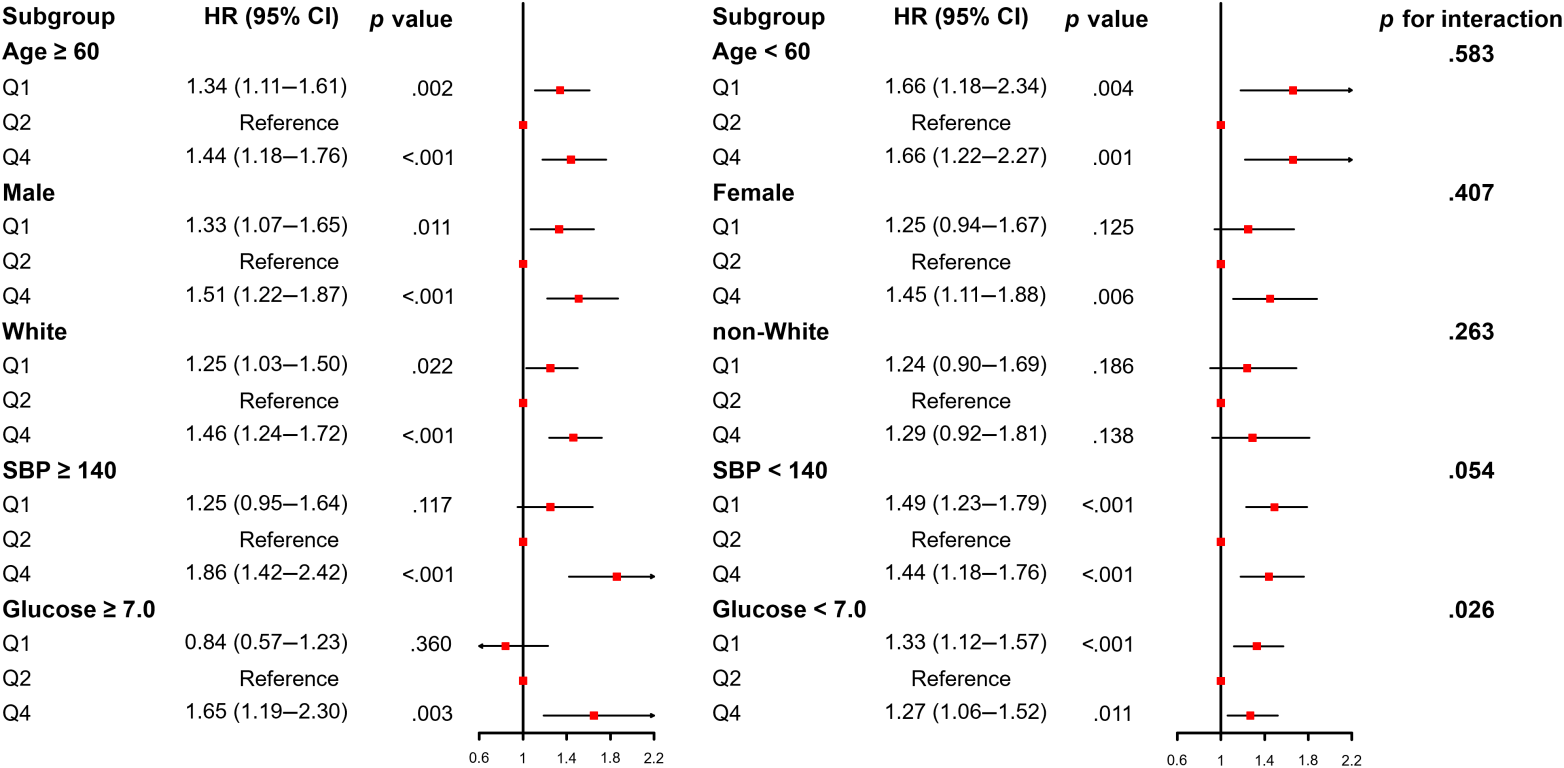
Subgroup analysis between SHR and all‐cause mortality stratified by age, sex, race or ethnicity, systolic blood pressure, and fasting glucose. HR, hazard ratio; SBP, systolic blood pressure; SHR, stress–hyperglycemia ratio.

The U‐shaped association between SHR and all‐cause mortality was similar across a wide range of subgroup participants stratified by age, sex, race/ethnicity, SBP, and FBG (Figure 4). Except for glucose (*p* for interaction = .026), no significant interaction was observed between stratified variables and SHR (all *p* for interaction >.05).

## 4 DISCUSSION

Based on 18 480 residents, our study explored the predictive value of the SHR associated with all‐cause mortality in the community‐dwelling population for the first time. Our findings indicated that SHR is significantly associated with all‐cause mortality in the community‐dwelling population. There was a U‐shaped relationship between SHR and all‐cause mortality. SHR and all‐cause mortality had a dose–response relationship, and the odds ratio (ORs) for all‐cause mortality was the lowest when the SHR index was 0.856.

### 4.1 The relationships of glycemic markers with all‐cause mortality in different populations

Previous studies have expounded a significant association between glycemic markers and all‐cause mortality in diabetics^37^, ^38^ and community dwellers.^39^, ^40^, ^41^ Elevated HbA1c, fructosamine, glycated albumin, and FBG were associated with mortality risk in older adults with diabetes.^37^

Universally known, increased values of HbA1c were associated with all‐cause and cardiovascular mortality in diabetes patients.^37^, ^42^, ^43^ In nondiabetic individuals, elevated HbA1c levels were associated with CVD incidence, overall mortality,^39^ and a linear association of HbA1c levels with primary cardiovascular events.^41^ A mostly monotonically increasing relationship was observed between HbA1c levels and outcomes with cardiovascular mortality, overall mortality, and CVD in the general European population.^39^ Besides, adults without diabetes with low HbA1c were at increased risk of all‐cause mortality.^40^ As such, the importance of HbA1c levels in the overall population is underlined.^39^

As for the relationships between FBG with all‐cause mortality, a study has shown low FBG is positively associated with all‐cause mortality risk in rural Chinese men but not in women.^44^ A J‐shaped relationship existed between FBG and all‐cause mortality and cardiovascular events in Korean older diabetes adults.^45^ Prediabetes, defined as impaired glucose tolerance, impaired fasting glucose, or raised HbA1c, was associated with an increased risk of cardiovascular disease.^46^ Whereas, in the nondiabetic population, increasing FBG is related to risks of MI, stroke, and all‐cause mortality, which is more rapid and more severe.^47^

Nevertheless, the accuracy of HbA1c used to assess average glucose has been controversial. Some experts question proposals to diagnose diabetes by raising glycated hemoglobin concentration rather than glucose testing.^48^ HbA1c may predict the risk of mortality and CV events in diabetes populations, but it is unlikely to be a strong predictor in patients without established diabetes.^49^ In a retrospective cohort of more than 200, 000 nondiabetic individuals, the risk model to predict CV events was not enhanced significantly by the addition of HbA1c.^50^ For cardiovascular and all‐cause mortality, the observed small effect sizes at both the lower and upper end of the HbA1c distribution do not support the notion of a J‐shaped association of HbA1c levels because a certain degree of residual confounding needs to be considered in the interpretation of the results.^41^ In addition, the phenomenon of glycemic variability exists in both the community population and diabetic patients. Variability of visit‐to‐visit FBG may be a more sensitive predictor of risk of death than mean‐FBG in type 2 diabetes.^51^ HbA1c may not be a robust marker of all‐cause mortality in patients with a high degree of glycemic variability.^38^ Although a new term, “A (1c)‐derived average glucose” is advocated to address the potential confusion^52^; evidence from several sources suggests that A (1c) is inherently imprecise as a measure of average glucose, so the proposed terminology should not be adopted.^53^ Low HbA1c and FBG may be markers of poor prognosis but are possibly confounded by health status.^37^

As such, there is a need for better glycemic markers to explore the relationships with all‐cause mortality in the community‐dwelling population. Previous studies have elaborated that SHR is closely associated with poor prognosis in patients with severe acute diseases,^2^ such as MI,^5^, ^6^, ^7^, ^8^ stroke,^9^, ^10^ and sepsis.^11^ The algorithm for SHR includes both HbA1c and glucose, which may more accurately reflect average glucose. Our study found a significant association between SHR and all‐cause mortality in community dwellers, which is largely consistent with previous research. The difference is that the subjects of our study are a community population, in whom SHR may be associated with potential psychological stress, various stress, and other factors. Better glycemic control is important for reducing mortality. New metrics of glycemic control may be needed in both diabetics and community dwellers.

### 4.2 The relationships of SHR with all‐cause mortality in community dwellers

Our study found a significant association between SHR and all‐cause mortality in community dwellers. Age, race or ethnicity, BMI, education level, marital status, economic status, smoking status, leisure time physically inactive, and almost all the laboratory measurements were significantly different among the four groups (all *p* < .001). We performed a multivariate Cox regression analysis correcting for these confounders (Table 2) and a subgroup analysis (Figure 4), which found consistent results across the different subgroups.

In general, stress is connected with a time‐limited initiation of the stress system, such as the hypothalamic–pituitary–adrenal (HPA) axis and the arousal/sympathetic nervous system.^22^ The influence of stressors is demonstrated by perturbations to physiological systems that are vital to homeostasis regulation and metabolic control.^3^ Therefore, measured stress indicators include the HPA‐derived hormone, cortisol, and the sympathoadrenal medullary mediators, epinephrine and norepinephrine, along with peripheral physiological indices such as heart rate and BP.^3^ Stress‐induced increases in glucagon, cortisol, and catecholamine release promote glycogenolysis and gluconeogenesis, eventually causing hyperglycemia.^54^ So in some situations, stress can be reflected in the SHR.^3^, ^4^

Stress‐induced inflammation may be adaptive, but chronic inflammation (a sustained stress situation) can contribute to processes like atherosclerosis, ultimately leading to the onset of CVD.^21^ In addition, biological mechanisms responsible for burnout are linked to sustained hyperactivation of the HPA axis.^20^ In the early stages, this adaptive response prepares for the “struggle” and has a potent anti‐inflammatory effect, preventing disease behaviors and reducing infection risk.^21^ Over time, this response manifests as psychological exhaustion and predisposes to various pro‐inflammatory diseases like metabolic syndrome, chronic pain, or CVD, along with a sickness behavior including psychomotor retardation, fatigue, or depression.^21^ Furthermore, chronic stress may be associated with persistent oversecretion or hyposecretion of stress system mediators, which is associated with increased long‐term mental and physical morbidity.^22^

Thus, the SHR can reflect the degree of stress (such as work stress, life stress, and emotional stress) to some extent and is significantly associated with all‐cause mortality in community dwellers. But it is physiological reactions that support adaptive behavior over the short term, although they can be maladaptive and generate disease in the long term.^3^ Thus, higher SHR in the community‐dwelling population is associated with increased all‐cause mortality over the long term.

### 4.3 The dose–response relationships of SHR with all‐cause mortality

The relationship between SHR and all‐cause mortality in the community‐dwelling population is U‐shaped (Figure 2). Specifically analyzing, the relationship of SHR with CVD mortality and cerebrovascular diseases is L‐shaped, whereas the relationship between SHR and renal diseases is J‐shaped. However, a positive association of SHR with malignant neoplasms and Alzheimer's disease and an inverse association between SHR and chronic lower respiratory diseases were observed (Figure S1).

A dose–response association was observed between SHR and all‐cause mortality in the RCS analysis. The highest risk for all‐cause mortality risk was observed when SHR ≤0.843 (Q1) or SHR ≥0.986 (Q4) (log‐rank *p* < .001). In addition, the second SHR quartile (Q2) has the best survival probability from Kaplan–Meier survival curves of all‐cause mortality.

On the one hand, there may be several reasons why an increase in SHR was associated with an increased risk of all‐cause mortality in community dwellers. First, higher SHR may mean the community‐dwelling population has been exposed to more stress recently, including work stress,^20^, ^21^, ^55^ life stress,^22^ and emotional stress.^18^ Previous study indicates work stress promotes and maintains metabolic syndrome and CVD.^21^ Longitudinal studies suggest that not only depression but general emotional stress and anxiety, sleeping problems, anger, and hostility contribute to an increased risk for the onset of type 2 diabetes.^18^ Second, psychological stress is one of the contributing factors to increasing all‐cause mortality in those with higher SHR in community dwellers. Psychological distress would be considered a risk factor for diabetes mellitus, may be detrimental to maintaining glycemic control, and could even lead to the pathogenesis of type 2 diabetes.^56^ Furthermore, mental stress caused greater ischemia than exercise in a small percentage of patients (20%).^57^ Third, higher SHR may indicate that community dwellers themselves have hyperglycemia or abnormal glucose metabolism, which promotes and maintains metabolic syndrome,^6^, ^58^ cardiovascular disease,^13^, ^55^ and other diseases.^9^ Disorders associated with metabolic syndrome are nearly always a proinflammatory state related to altered glucose metabolism, which could increase cardiovascular risk.^59^

On the other hand, there may be two main reasons that decreased SHR was associated with an increased risk of all‐cause mortality. For one thing, the adaptive stress response may not be effectively activated, so the individual cannot effectively prepare for the “struggle.”^21^ For another, low SHR may mean low psychosocial stress, which may elevate the risk of type 2 diabetes and numerous complications. Previous study indicates low emotional support may augment the risk of type 2 diabetes in women.^55^

Therefore, both higher SHR and lower SHR in the community‐dwelling population are associated with increased all‐cause mortality. However, the relationship between glucose concentrations and patient outcomes is complex. Although our data demonstrate that SHR could provide prognostic information in community‐dwelling populations, for they are under various stresses. Further studies are needed to verify the predictive value and clinical significance.

### 4.4 Use fasting blood glucose to calculate SHR

The formula uses acute glucose, but the community population is not necessarily under acute stress; they may be under chronic stress. Besides, the blood drawn from the community population is mostly collected on an empty stomach, and few people test glucose in the acute state. Therefore, FBG can also reflect the glucose under psychological stress and various stresses of the community population.

Stress hyperglycemia is a complex phenomenon, the cumulative effect of a combination of a variety of factors. The rapid change of glucose may reflect neurohormonal and homodynamic events that influence patient prognosis.^59^

As the majority of the community‐dwelling population is expected to be “healthy,” the proportion of those unhealthy in this population is lower than in the general population. In our study, we use FBG instead of RBG.

Port et al first supposed that the use of RBG rather than FBG might introduce additional random error on account of variability in sampling time.^60^ What is more, it has been recommended not to use “RBG” for CVD risk assessment by reason of blood glucose levels obtained at different sampling times have different meanings. Einarson et al conducted a meta‐analysis and observed significant relationships between blood glucose (fasting, 2‐h postprandial, 1‐h postprandial or random) and the total number of cardiovascular events (MI, stroke, death) and cardiovascular deaths in people without diabetes.^61^ In the situation of cardiac death, in addition to the RBG, in all cases a significant relationship was observed.^61^

Thus, the calculation of SHR with FBG is more accurate and suitable for the community‐dwelling population. In the subgroup analysis, though significant interaction was observed between glucose and SHR (*p* for interaction = .026), a similar U‐shaped relationship of glucose between glucose and all‐cause mortality was observed. The reason may be the effect of sample size between subgroups.

### 4.5 Strengths and limitations

The strength of this study is that the study cohort of the community‐dwelling population was derived from a national registry‐level database, which allowed comprehensive and accurate case capture. This is the first report of a dose–response relationship of SHR with all‐cause mortality in the community‐dwelling population using RCS analysis.

However, we recognize several limitations of this study. First, we could not exclude the probability of selection bias given that over half the patients in the database were excluded from the analysis owing to missing data. To reduce selection bias, we have additionally compared basic demographics and other characteristics between those with SHR and without SHR, which indicated nearly no significant difference (Table **S1**). Second, because this was a retrospective study, causality cannot be determined in this study; further prospective multicenter studies are needed to validate these results. Third, we could standardize only the time of the fasting glucose levels (after an overnight fast of 8 h) as we used retrospective data, though this does reflect real‐world practice.

## 5 CONCLUSIONS

The SHR was significantly associated with all‐cause mortality in the community‐dwelling population, and there was a U‐shaped relationship.

## AUTHOR CONTRIBUTIONS

Conceptualization: Shifeng Qiu, Xiaocong Liu, and Li Lei; Methodology: Shifeng Qiu, Xiaocong Liu, and Li Lei; Validation: Hongbin Liang and Xue Li; Formal analysis: Juefei Wu, Yuegang Wang, and Daogang Zha; Investigation: Yutian Wang, Chen Yu, Xiaobo Li, and Yongzhen Tang; Data curation: Xuewei Liu and Min Xiao; Writing – original draft preparation: Shifeng Qiu, Xiaocong Liu, and Li Lei; Writing – review and editing: Hongbin Liang, Xue Li, Yutian Wang, Chen Yu, Xiaobo Li, Yongzhen Tang, Juefei Wu, Yuegang Wang, Daogang Zha, Xuewei Liu, Min Xiao, and Jianchen Xiu; Visualization: X.L.; Supervision: Min Xiao and Jianchen Xiu; Funding acquisition: Jianchen Xiu. All authors have read and agreed to the published version of the manuscript. All authors have read and agreed to the published version of the manuscript.

## FUNDING INFORMATION

This research was funded by the National Natural Science Foundation of China (81974266), and the Guangzhou Key Research and Development Program (202206080014).

## CONFLICT OF INTEREST STATEMENT

The authors declare no conflict of interest.

## INSTITUTIONAL REVIEW BOARD STATEMENT

The study was conducted according to the guidelines of the Declaration of Helsinki. The studies involving human participants were reviewed and approved by the Ethics Committee of the Nanfang Hospital.

## INFORMED CONSENT STATEMENT

Informed consent was obtained from all subjects involved in the study.

## Supporting information


**Figure S1.** Association between SHR and cause‐specific death.

**Table S1.** Subgroup analysis between SHR and cause‐specific mortality.

## Data Availability

Publicly available datasets were analyzed in this study. The data can be found here: [https://wwwn.cdc.gov/nchs/nhanes/].
