# Supplementary figures and images for: Association between the stress–hyperglycemia ratio and all‐cause mortality in community‐dwelling populations: An analysis of the National Health and Nutrition Examination Survey (NHANES) 1999–2014
Source: J Diabetes. 2024 May 20;16(6):e13567. doi: 10.1111/1753-0407.13567 (PMC11106591; doi:10.1111/1753-0407.13567)

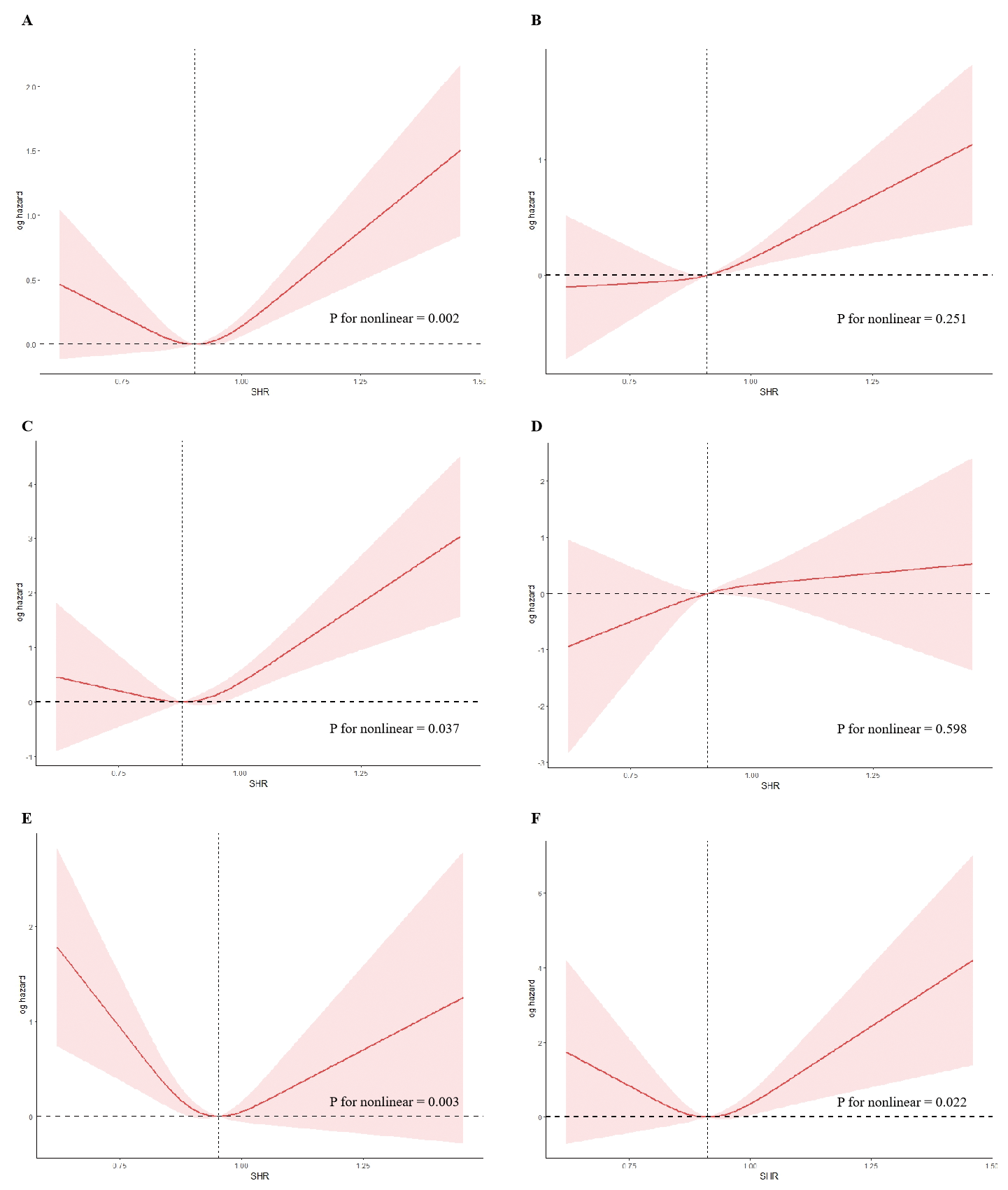

Supplement: Supplementary file 1 — Figure S1. Association between SHR and cause‐specific death. [file JDB-16-e13567-s001.tif]
