# Supplementary material for: Association between the stress–hyperglycemia ratio and all‐cause mortality in community‐dwelling populations: An analysis of the National Health and Nutrition Examination Survey (NHANES) 1999–2014
Source: J Diabetes. 2024 May 20;16(6):e13567. doi: 10.1111/1753-0407.13567 (PMC11106591; doi:10.1111/1753-0407.13567)
Supplement: Supplementary file 2 — Table S1. Subgroup analysis between SHR and cause‐specific mortality. [file JDB-16-e13567-s002.docx]

Supplementary Material

**Table S1.** Subgroup analysis between SHR and cause-specific mortality.

|  | Adjusted Model * | |
| --- | --- | --- |
|  | HR (95% CI) | P value |
| CVD mortality | | |
| Q1 | 1.65 (1.18-2.31) | 0.004 |
| Q2 | Reference |  |
| Q3 | 1.55 (1.12-2.16) | 0.009 |
| Q4 | 1.75 (1.28-2.40) | <0.001 |
| Malignant neoplasms | | |
| Q1 | 1.19 (0.85-1.66) | 0.316 |
| Q2 | Reference |  |
| Q3 | 1.54 (1.13-2.11) | 0.007 |
| Q4 | 1.53 (1.14-2.03) | 0.004 |
| Cerebrovascular diseases | | |
| Q1 | 1.22 (0.64-2.33) | 0.555 |
| Q2 | Reference |  |
| Q3 | 1.48 (0.76-2.89) | 0.251 |
| Q4 | 2.26 (1.25-4.12) | 0.007 |
| Alzheimer's disease | | |
| Q1 | 0.64 (0.33-1.24) | 0.186 |
| Q2 | Reference |  |
| Q3 | 0.99 (0.45-2.15) | 0.978 |
| Q4 | 1.01 (0.36-2.82) | 0.988 |
| Chronic lower respiratory diseases | | |
| Q1 | 1.34 (0.89-2.01) | 0.167 |
| Q2 | Reference |  |
| Q3 | 1.23 (0.83-1.81) | 0.300 |
| Q4 | 0.68 (0.43-1.07) | 0.095 |
| Renal diseases | | |
| Q1 | 2.83 (0.59-13.70) | 0.195 |
| Q2 | Reference |  |
| Q3 | 1.84 (0.38-8.94) | 0.450 |
| Q4 | 3.82 (1.06-13.82) | 0.041 |

*adjusted for age, sex, race/ethnicity, body mass index, education levels, marital status, economic status, smoking status, alcohol drinking status, leisure time physical inactive, systolic blood pressure, diastolic blood pressure, hemoglobin, total cholesterol, high-density lipoprotein cholesterol, uric acid, alanine transaminase, estimated glomerular filtration rate, self-reported cardiovascular disease using appropriate sampling weights.

**Supplementary Materials:**


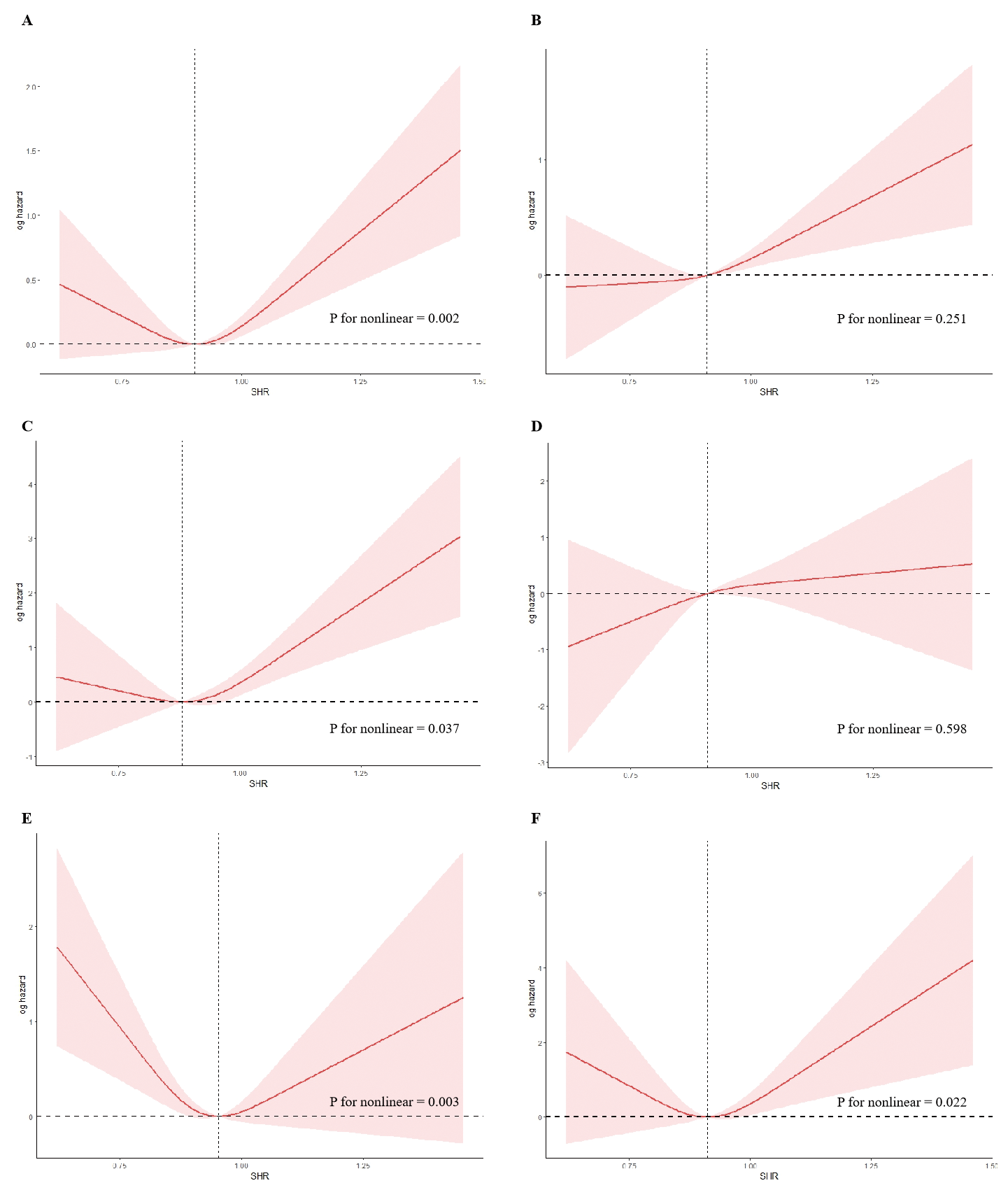


**Figure S1.** Association between SHR and cause-specific death. A. CVD mortality; B. Malignant neoplasms; C. Cerebrovascular diseases; D. Alzheimer's disease; E. Chronic lower respiratory diseases; F. Renal diseases.
